# Goat K_222_-PrP^C^ polymorphic variant does not provide resistance to atypical scrapie in transgenic mice

**DOI:** 10.1186/s13567-016-0380-7

**Published:** 2016-09-22

**Authors:** Patricia Aguilar-Calvo, Juan-Carlos Espinosa, Olivier Andréoletti, Lorenzo González, Leonor Orge, Ramón Juste, Juan-María Torres

**Affiliations:** 1 Centro de Investigación en Sanidad Animal (CISA-INIA), Valdeolmos, Madrid, Spain; 2 Interactions Hôte Agent Pathogène, École Nationale Vétérinaire de Toulouse, Toulouse, France; 3 Animal and Plant Health Agency, Penicuik, Midlothian, UK; 4 Instituto Nacional de Investigação Agrária e Veterinária, Lisbon, Portugal; 5 Neiker-Tecnalia, Derio, Vizcaya Spain; 6 Department of Pathology and Medicine, University of California San Diego (UCSD), La Jolla, USA

## Abstract

Host prion (PrP^C^) genotype is a major determinant for the susceptibility to prion diseases. The Q/K_222_-PrP^C^ polymorphic variant provides goats and mice with high resistance against classical scrapie and bovine spongiform encephalopathy (BSE); yet its effect against atypical scrapie is unknown. Here, transgenic mice expressing the goat wild-type (wt) or the K_222_-PrP^C^ variant were intracerebrally inoculated with several natural cases of atypical scrapie from sheep and goat and their susceptibility to the prion disease was determined. Goat wt and K_222_-PrP^C^ transgenic mice were 100% susceptible to all the atypical scrapie isolates, showing similar survival times and almost identical disease phenotypes. The capacity of the K_222_-PrP^C^ variant to replicate specifically the atypical scrapie strain as efficiently as the goat wt PrP^C^, but not the classical scrapie or cattle-BSE as previously reported, further suggests the involvement of concrete areas of the host PrP^C^ in the strain-dependent replication of prions.

## Introduction

Scrapie is a fatal neurodegenerative disease of sheep and goats caused by the conversion of the host cellular prion protein (PrP^C^) into a pathological misfolded form (PrP^Sc^). Scrapie occurs in a variety of phenotypes within two forms—classical and atypical scrapie forms—which differ in their incubation periods, clinical signs, neuropathological lesion profiles and/or PrP^Sc^ biochemical properties. The zoonotic potential of classical scrapie has been recently proposed from transmission studies of a panel of classical scrapie isolates in non-human primates [1] and in humanized transgenic mice [2], highlighting the necessity of deciphering the factors involved in prion transmission.

Susceptibility of sheep and goats to scrapie is strongly determined by the PrP^c^ encoding gene (*Prnp*) and the prion strain. Strategies to promote breeding for the *Prnp* allele linked to classical scrapie resistance, A_136_R_154_R_171_, in sheep herds were implemented in some European countries and in the USA, resulting in rapid decline of classical scrapie outbreaks [3]. Unfortunately, A_136_R_154_R_171_ genotype does not provide resistance towards atypical scrapie [4].

We have previously demonstrated the high resistance of the goat K_222_-PrP^C^ variant to a wide variety of classical scrapie isolates using transgenic (Tg) mice [5]. These results were confirmed by oral and intracerebral inoculations of a natural classical scrapie isolate in goats expressing this polymorphism [6] and we therefore proposed the K_222_-PrP^C^ variant as a good candidate for developing selective breeding programs in goat herds. These studies are very valuable to control the spread of prion diseases in animals, but also provide crucial information about the role of the host genotype in the prion pathobiology. In the present study, we analyze the role of K_222_-PrP^C^ variant on the resistance/susceptibility of goats to atypical scrapie using goat-PrP Tg mice.

## Materials and methods

### Ethics statements

Animal experiments were carried out in strict accordance with the recommendations of the Code for Methods and Welfare Considerations in Behavioral Research with Animals (Directive 86/609EC) and approved by the Committee on the Ethics of Animal Experiments (CEEA) of the Spanish Instituto Nacional de Investigación y Tecnología Agraria y Alimentaria (INIA); Permit Number: CEEA2011/050.

### Transmission studies

Groups of 5–6 Tg mice (6–7 weeks old) expressing either the goat wild type (wt) PrP^C^ (Q_222_-Tg501) or the K_222_-PrP^C^ variant (K_222_-Tg516) at two fold the PrP^C^ levels in the goat brain [5] were intracerebrally inoculated into the right parietal lobe with 20 μL of 10% brain homogenate (w/v) from sheep and goats naturally infected with atypical scrapie (see Table 1 for isolate information). Mice were monitored daily and their neurological status assessed twice a week. Mice were humanely euthanized when the progression of the disease was evident or at the end of their life span [≈650 days post-inoculation (dpi)]. During necropsy, brains were sliced sagittally and one hemibrain was fixed in 10% buffered formalin for histopathological analysis. The remaining hemibrain was homogenized as 10% (w/v) for the detection of proteinase K-resistant PrP (PrP^res^) by Western blot. Survival time was calculated as the mean number of survival dpi for all the mice positive for PrP^res^ in the brain, with the standard error included, while attack rate was expressed as the proportion of PrP^res^-positive mice among the mice inoculated.

**Table 1:** **Description of natural atypical scrapie isolates**

Isolate	PrP genotype	Description and references	Supplier
Sheep-Sc M45	wt^a^	Cerebellum from a natural atypical scrapie case in sheep	NEIKER^c^
Goat-Sc I15	wt^b^	Brain from a natural atypical scrapie case in goat	IZSTO^d^
Goat-Sc 12-09106	wt; H/H_154_	Cerebellum from a natural atypical scrapie case in goat	INIAV^e^

^a^Wild type (wt) sheep prion protein genotype: A_136_R_154_Q_171_/A_136_R_154_Q_171._

^b^Wild type (wt) goat prion protein genotype: I_142_R_154_R_211_Q_222_S_240_/I_142_R_154_R_211_Q_222_S_240._

^c^Instituto Vasco de Investigación y Desarrollo Agrario (NEIKER), Vizcaya, Spain.

^d^Istituto Zooprofilattico Sperimentale de Piemont, Torino (IZSTO) Italy (We than Dr. Pier Luigi Acutis for providing the Goat-Sc I15 isolate).

^e^Laboratório de Patologia; Instituto Nacional de Investigação Agrária e Veterinária (INIAV); Lisboa, Portugal.

### Western blotting

Brain tissue was homogenized in 5% glucose in distilled water in grinding tubes (Bio-Rad) and adjusted to 10% (w/v) by using a TeSeETM Precess 48TM homogenizer (Bio-Rad) following the manufacturer’s instructions. To determine the presence of PrP^res^ in transgenic mouse brains, 100 µL of 10% brain homogenate were analyzed by Western blotting as previously described [7] with some modifications. Briefly, digestion was done with 40 µg/mL of proteinase K in buffer 5% sarkosyl, 5% Triton X100, 1 M Urea and 16 mM Tris–HCl (pH 9.6) at 60 °C for 15 min. Samples were electrophoresed in 12% Criterion XT Bis–Tris Gel (BioRad). For immunoblotting, membranes were incubated with 12B2 PrP monoclonal antibody (epitope _93_WGQGG_97_ of the sheep PrP sequence) at a final concentration of 1 μg/mL. Immunocomplexes were detected with horseradish peroxidase-conjugated anti-mouse IgG (Amersham Pharmacia Biotech) after incubating the membranes for 1 h, and blots were developed with chemiluminescent substrate ECL Select (GE Healthcare Amersham Biosciences). Images were captured using ChemiDoc XRS + System and then processed using Image Lab 5.2.1 Software.

### Histopathological analysis

Formalin-fixed brains were trimmed, dehydrated, embedded in paraffin-wax, cut to 4 µm thickness, de-waxed and rehydrated by standard procedures. For IHC demonstration of PrP^Sc^ accumulation, tissue sections were subjected to antigen retrieval and quenching of hydrogen peroxide, as described previously [8] and incubated with polyclonal PrP antibody R486 (APHA, Weybridge, UK; _240_SPPVILLISFLIFLI_254_ epitope of the sheep PrP sequence); the subsequent steps of the IHC procedure were performed by a commercial immunoperoxidase technique (Vector-Elite ABC kit, Vector Laboratories) as per manufacturer’s instructions and sections were finally counterstained with Mayer’s haematoxylin. The PrP^Sc^ types considered included the intraneuronal (ITNR), intraglial (ITGL, intra-microglial and intra-astrocytic combined), stellate (STEL), fine particulate (PART), coalescing (COAL), linear (LINR) and plaques (PLAQ, vascular and non-vascular combined) as described elsewhere [8]. Plaques were only considered as such when they showed a homogeneous core and a radiate periphery (mature plaques), while in the absence of these features the dense accumulations of PrP^Sc^ were termed “coalescing”, without prejudice to them actually corresponding to primitive plaques.

## Results

Tg mouse lines expressing the goat wild type PrP^C^ (Q_222_-Tg501) and the K_222_-PrP^C^ variant (K_222_-Tg516) were intracerebrally challenged with 3 natural cases of atypical scrapie (Table 1) and their susceptibilities assessed and compared following previously described methods [5].

Both Q_222_-Tg501 and K_222_-Tg516 mice succumbed to the inoculation of all atypical scrapie isolates with 100% attack rates (Table 2). For each isolate, Q_222_-Tg501 and K_222_-Tg516 mice displayed similar survival times which were longer than 300 dpi in all cases (Table 2).

**Table 2:** **Transmission of atypical scrapie to Q**_**222**_ **-Tg501 and K**_**222**_ **-Tg516 mice**

Isolate	Mean survival time in days ± SD (n/n_0_)	
	Q_222_-Tg501	K_222_-Tg516
Sh-Sc M45	443 ± 70 (9/9)	441 ± 131 (10/10)
Goat-Sc I15	552 ± 78 (6/6)	552 ± 64 (6/6)
Goat-Sc 12-09106	331 ± 15 (7/7)	309 ± 34 (6/6)

*n/n*_*0*_ attack rate expressed as the proportion of positive mice among the total inoculated mice

**Figure 1:**
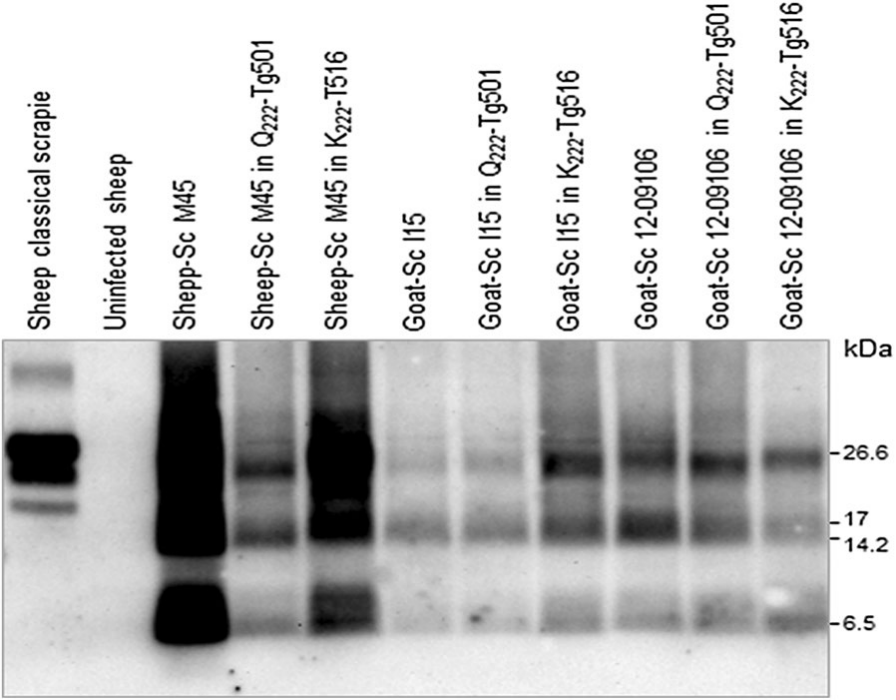
**Brain PrP** ^**res**^ **from Q**_**222**_ **-Tg501 and K**_**222**_ **-Tg516 mice inoculated with an atypical scrapie isolate.** Immunoblots of brain PrP^res^ detected with 12B2 mAb of atypical scrapie isolates before and after transmission in either Q_222_-Tg501 or K_222_-Tg516 mice. Sheep classical scrapie and uninfected sheep brain were included on the right. Similar amounts of 10% brain homogenate were loaded in each lane for comparison. Both Q_222_-Tg501 and K_222_-Tg516 mice showed a similar PrP^res^ ladder pattern characteristic of atypical scrapie. Molecular masses (kDa) are shown on the right.

PrP^res^ was detected in the brains of all the inoculated Q_222_-Tg501 and K_222_-Tg516 mice by Western blot. PrP^res^ glycoprofiles were characterized by a ladder-like pattern and a low unglycosylated band of around 7 kDa similar to that observed in their respective original sheep or goat isolates (Figure 1).

**Figure 2:**
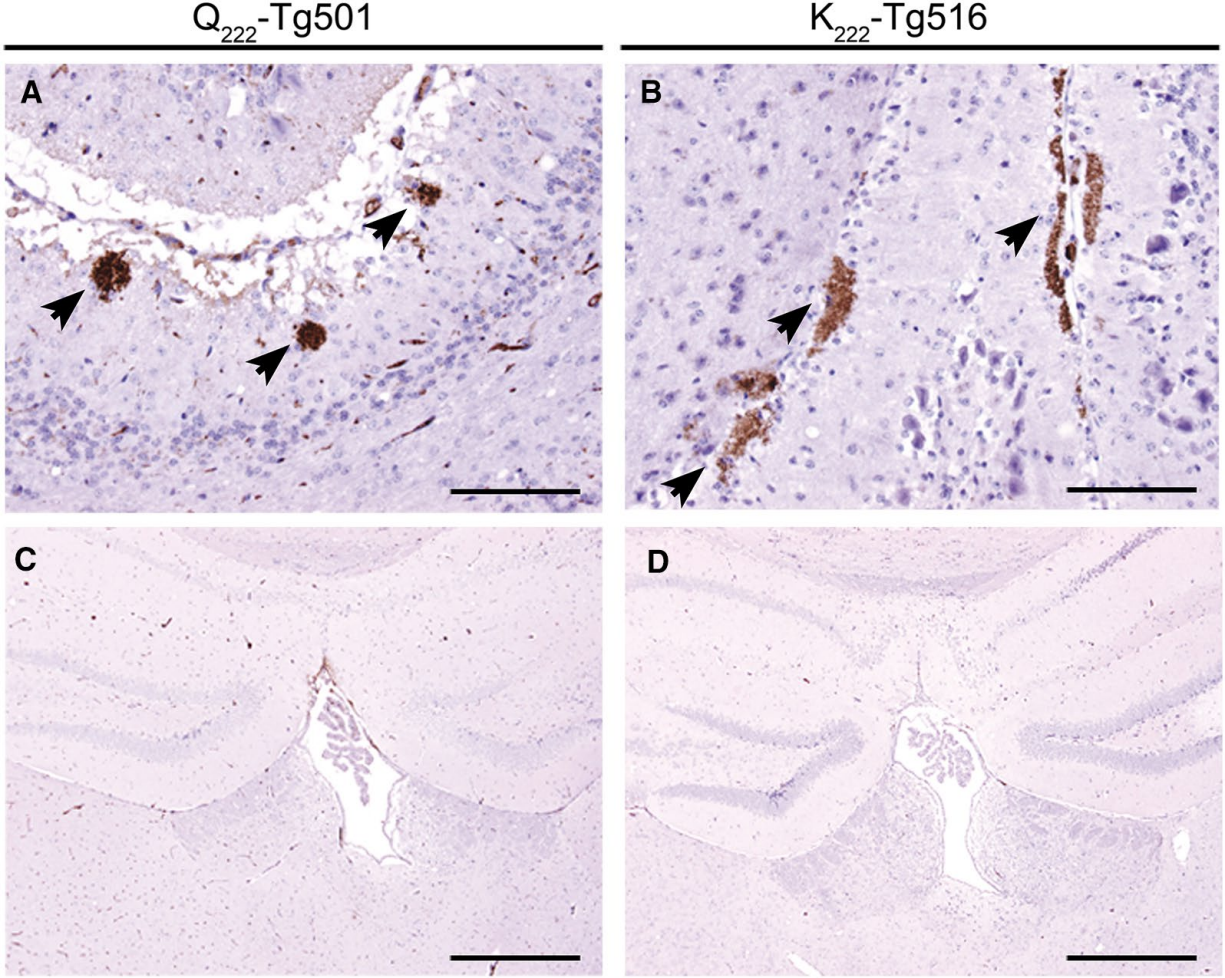
**PrP** ^**Sc**^ **accumulation in the brain of Q**_**222**_ **-Tg501 and K**_**222**_ **-Tg516 mice infected with Sheep-Sc M45.** Immunohistochemistry with R486 PrP antibody and haematoxylin counterstaining. PrP^Sc^ remained located to the molecular layer of the cerebellum as amorphous coalescing or plaque-like deposits (arrows) (**A**, **B**). Note the absence of PrP^Sc^ and vacuolation in the hippocampus and dorsal thalamus (**C**, **D**). Scale bars are 100 µm (**A**, **B**) and 500 µm (**C**, **D**).

All infected Q_222_-Tg501 and K_222_-Tg516 mice showed PrP^Sc^ in their brains with indistinguishable immunohistochemical phenotypes. PrP^Sc^ appeared as occasional, scattered coalescing or amorphous plaque-like deposits restricted to the molecular layer of the cerebellum while vacuolation was minimal in this area (Figure 2A, B). No PrP^Sc^ deposition or spongiform change were observed in any of the other brain areas examined, as exemplified by the hippocampus and thalamus in Figure 2C and D.

## Discussion

Intracerebral inoculations of prions in transgenic mice expressing different PRNP polymorphic variants are highly valuable to point to a specific amino acid substitution as the unique mutation responsible for the resistance/susceptibility of a species to a prion disease. Our results indicate that K_222_-PrP^C^ variant replicates the atypical scrapie agent as efficiently as the wt-PrP^C^ and thus, it might not be able to protect goats against the infection with this prion disease. However, this assumption must be confirmed by oral inoculation of Q/K_222_ goats since there is not always a correlation between intracerebral inoculations in transgenic mouse models and field infections in natural hosts [9].

Intracerebral transmission of atypical scrapie in both Q_222_-Tg501 and K_222_-Tg516 mouse lines resulted in a prion disease characterized by noticeable long survival times (>300 dpi) and poor pathology in brain. These long survival times are consistent with the old age at which sheep and goats normally develop atypical scrapie either naturally (over 5 years of age) [4] or after intracerebral transmission (around 2.5 years of age) [10] and suggests that atypical scrapie is a slow infectious agent in its natural PrP^C^ species context. In line with this view, mice overexpressing 8- to 10-fold the sheep PrP^C^ in their brains succumbed to the intracerebral inoculation of multiple French atypical scrapie isolates only after more than 400 dpi [11]. However, prolonged survival times are not exclusive of atypical scrapie agents since particular classical scrapie isolates can produce survival times longer than 500 dpi even after serial intracerebral passages in homozygous Q_222_-Tg501 mice (unpublished data).

The histological and immunohistochemical examinations of the brains from all the atypical scrapie infected Q_222_-Tg501 and K_222_-Tg516 mice consistently revealed a mild accumulation of PrP^Sc^ restricted to the cerebellum with little or no vacuolation also limited to this brain area. These outcomes clearly contrast with the widespread PrP^Sc^ and intense vacuolation observed in classical scrapie infected Q_222_-Tg501 mice or in BSE infected K_222_-Tg516 mice [5]; instead they correlate well with the particularly prominent PrP^Sc^ deposition in the cerebellum of sheep and goats with natural atypical scrapie [12]. The PrP^res^ glycoprofiles of all the atypical scrapie isolates were also maintained in both Q_222_-Tg501 and K_222_-Tg516 mouse lines which, together with the survival times and the histological outcomes, further demonstrates the validity of our animal models to characterize prion strains of sheep and goats.

Susceptibility to prions and disease phenotypes are likely modulated by conformational properties of prion strains and the PrP^C^ and PrP^Sc^ amino acid sequence [13]. Some host PrP^C^ amino acid sequences may have greater “plasticity” or ability to misfold—as proposed for the unique susceptibility of bank voles to a wide variety of prion diseases [14] and to “in vitro” conversion experiments [15]—while others may show the opposite situation, locking PrP^C^ and preventing its conversion by different prion strains. Nonetheless, the plasticity of the host PrP^C^ would not explain why certain amino acid substitutions inhibit the replication of some prion strains but not others. A clear example is the goat K_222_ variant which provides high resistance against classical scrapie and bovine spongiform encephalopathy (BSE) from cattle—being protective even in heterozygous mice and goats [5, 6, 9]—but it is as efficient as the goat wt PrP^C^ in replicating sheep and goat-BSE [5] and atypical scrapie when intracerebrally inoculated. The resistance of the K_222_ variant to classical scrapie was associated to the additional positive charge at codon 222 provided by the lysine amino acid, which could interfere with the PrP^C^:PrP^Sc^ interaction, abolishing or lowering the conversion rates of PrP^C^ [16]. Similarly, the perturbations in the PrP^C^ surface charge distribution and structural rearrangements mainly localized at the β2–α2 loop region (residues 169–179 in goat PrP^C^ numbering) are likely to underlie the resistance of the human E/K_219_ variant against CJD [17].

More recently, the existence of critical initial PrP^C^–PrP^Sc^ interaction sites during the templating of PrP^C^ by different prions was proposed [18]. The involvement of specific PrP^C^ areas in the strain-dependent replication of prions could account for the variable role of the K_222_-PrP^C^ variant in the susceptibility to scrapie. While for classical scrapie polymorphic variants along the PrP^C^ have been associated to modulate the susceptibility to the disease, for atypical scrapie only few have been reported—A/V_136_, F/L_141_, R/H_154_ and Q/R_171_ [4, 19]— that, interestingly, are all located within β1–α1 and β2–α2 loops. This area could be the critical segment for atypical scrapie interaction with the host PrP^C^ and therefore any amino acid exchange outside this segment would not affect the capacity of replication of the atypical scrapie, as observed in our transmission study in goat wt and K_222_-PrP^C^ mice. In fact this area seems to be accessible in the abnormally folded aggregate of the atypical scrapie agent as suggested by the observation that the C-terminal part of atypical scrapie PrP^Sc^ can be specifically trimmed by PK [20]. In conclusion, although more information is still needed to decipher the mechanisms of prion conversion, our study points to the involvement of specific PrP^C^ areas in the strain-dependent replication of prions. Thus, strategies to fight prion diseases based on genotype breeding programs should be prion strain targeted.
